# Optimal Reference Gene Selection and Potential Target Gene Identification During *Xanthomonas phaseoli* pv. *dieffenbachiae*–*Anthurium andreanum* Infection

**DOI:** 10.3390/mps8040072

**Published:** 2025-07-04

**Authors:** Shu-Cheng Chuang, Shefali Dobhal, Teresita D. Amore, Anne M. Alvarez, Mohammad Arif

**Affiliations:** 1 Department of Plant and Environmental Protection Sciences, University of Hawaii at Manoa, Honolulu, HI 96822, USA; chuangsc@hawaii.edu (S.-C.C.); shefali@hawaii.edu (S.D.); alvarez@hawaii.edu (A.M.A.); 2 Department of Tropical Plant and Soil Sciences, University of Hawaii at Manoa, Honolulu, HI 96822, USA; amore@hawaii.edu

**Keywords:** *Xanthomonas phaseoli* pv. *dieffenbachiae*, anthurium bacterial blight, optimal reference gene, pathogenicity-related gene, qRT-PCR

## Abstract

*Xanthomonas phaseoli* pv. *dieffenbachiae* (Xpd), the causal agent of bacterial blight in *Anthurium* within the Araceae family, is listed as an EPPO A2 quarantine organism. Although the whole genome of Xpd has been sequenced, the molecular mechanisms underlying anthurium bacterial blight (ABB) remain unknown. Selecting an optimal reference gene is crucial for obtaining accurate and reliable gene expression profiles during the initial interactions between Xpd and *Anthurium*. The stability of four reference genes was evaluated by applying three statistical methods—BestKeeper, geNorm, and delta Ct (ΔCt)—using reverse-transcription quantitative PCR (RT-qPCR) data. The *rpoD* and *gyrB* genes exhibited the most consistent gene expression profiles, whereas *atpD* and *thyA* were less stable at four time points (0, 0.5, 1, and 2 h) during the interactions between Xpd and susceptible *A. andreanum* cultivar ‘Marian Seefurth.’ The suitability of these reference gene candidates was validated by normalizing the gene expression levels of four pathogenicity-related genes. The highly upregulated expression of *gumD*, which encodes xanthan biosynthesis glycosyltransferase, observed after 1 h of interaction, suggests it may be a key virulence determinant in the Xpd–*Anthurium* pathosystem. The stable reference genes identified here will facilitate more accurate and comprehensive gene expression studies in the Xpd–*Anthurium* pathosystem going forward.

## 1. Introduction

According to Constantin et al., *Xanthomonas phaseoli* pv. *dieffenbachiae* (Xpd) is listed an EPPO A2 quarantine organism and causes serious bacterial leaf blight on *Anthurium*, which belong to the Araceae family [1,2,3,4]. The *Anthurium* genus contains about 950 species, including one of the best-known—*A. andraeanum*—which is famous for its beautiful spathes shape and wide range of colors in the cut flower and potted ornamental markets [5,6]. *Anthurium* bacterial blight (ABB) is an economically important disease for which outbreaks have been reported in Hawaii and other tropical and subtropical growing countries worldwide [6,7,8,9,10,11]. The symptoms of ABB include water-soaking spots near the margins of the leaf, chlorosis, and necrotic zones following coalescence, systemic infection spreading to petiole and stem, senescence and distortion, and, ultimately, the blackening and decay of the plant [10,12]. Xpd could be spread through aerosol, rain, irrigation water, infected plant material, and contaminated tools, potentially causing a 50–100% crop loss once ABB is introduced into a new anthurium farm [6].

The whole genome of Xpd has been deciphered, revealing numerous pathogenicity factors, including extracellular polysaccharides (EPSs); lipopolysaccharides (LPSs); cell-wall-degrading enzymes (CWDEs); type II, III, IV, and VI secretion systems (T2, 3, 4, 6SS); and the T3 secretion effector (T3SE) repertoire. These factors, which are present in the genomes of LMG 695 pathotype and D182 strains [11,13], have shown the potential to facilitate systemic infection in *Anthurium* and *Dieffenbachia*. However, the relationship between these pathogenicity factors and host specificity remains underexplored, despite the identification of some unique genes and clusters such as T3SE *XopAO* and various LPS gene clusters in Xpd [10,13]. Global gene expression analysis should be employed to elucidate the molecular mechanisms underlying the interactions between Xpd and *Anthurium*.

Reverse-transcription quantitative PCR (RT-qPCR) is a rapid, reproducible, and highly sensitive and specific method that is widely used to investigate target gene expression under various natural conditions and/or experimental treatments [14]. To obtain a reliable analysis of an RT-qPCR assay, an optimal reference gene is essential as an internal control to normalize mRNA data [15]. Since there is no universal reference gene suitable for all experimental designs, a preliminary analysis is required to identify the most appropriate reference gene for the specific tissue, cell type, and conditions [15].

In previous studies, several reference genes in xanthomonads were examined under different conditions of in vivo and in vitro experiments. Reference genes—*thyA* (thymidylate synthase) and *gyrB* (DNA gyrase subunit B)—were validated as the optimal reference genes in *X. campestris* pv. *campestris* when grown on *hrp*-inducing medium (MMXC) [16]. Meanwhile, *atpD* (ATP synthase subunit δ) and *rpoD* (RNA polymerase δ factor) were identified as the most stably expressed genes in *X. arboricola* pv. *juglandis* under three abiotic stresses [17]. Additionally, under another pathosystem between *X. citri* subsp. *citri* and sweet orange, *atpD*, *rpoB* (RNA polymerase subunit β), *gyrA* (DNA gyrase subunit A), and *gyrB* were the most stable reference genes among the nine candidates evaluated [18]. Lastly, along with the *gyrB* gene, *ffh* (signal recognition particle protein) and *pykA* (pyruvate kinase) were the most appropriate internal controls in *X. fragariae* on strawberry [19].

The objective of this study was to identify the ideal normalizers in the interactions between Xpd and *Anthurium*. Based on previous studies, four reference gene candidates—*atpD*, *gyrB*, *rpoD*, and *thyA*—were selected to test the suitability. The stability of these genes was evaluated using RT-qPCR data generated from the time course samples, employing popular methods such as the BestKeeper [20], geNorm [21], and delta Ct (ΔCt) methods [22]. The gene expression profiles of four pathogenicity-related target genes—*hrpG* and *hrpX* (encoding master regulators of the type III secretion system (T3SS)-specialized syringe that injects effectors into host cells to suppress defenses and promote infection), *fliM* (encoding a key component of flagellar biosynthesis—critical for motility, initial host colonization, and biofilm formation), and *gumD* (responsible for exopolysaccharide xanthan production) were normalized using the appropriate reference genes, revealing the potential determinants of virulence or pathogenicity in Xpd during its infection of *A. andreanum* [23].

## 2. Materials and Methods

### 2.1. Bacterial Strain and Plant Materials

*Xanthomonas phaseoli* pv. *dieffenbachiae* (Xpd) strain PL36, isolated from Hawaii in 2016 [9], was used to identify optimized reference genes. The strain was streaked out from −80 °C glycerol stock and cultured on Nutrient agar (NA) media at 28 °C for two days. The *Anthurium* microplant planting method was adapted from the protocol described by Ayin et al. [8] with some modifications. Briefly, a susceptible *A. andreanum* cultivar ‘Marian Seefurth’ (MS) was cultured in vitro for 4–6 weeks. After rooting, the plants were deflasked and transferred into humidity domes filled with orchid bark (Daltons Ltd., Matamata, New Zealand) for another 4–6 weeks with full spectrum plant growing LED light (Feit Electric, Pico Rivera, California, USA) for 12 h daily at 24–26 °C.

### 2.2. Selection of Reference Genes, Target Genes, and Primer Design

Four housekeeping genes–*atpD*, *gyrB*, *rpoD*, and *thyA*–were selected to demonstrate the optimal reference gene for Xpd-*Anthiurim* interaction. In order to verify the accuracy and practicability, four pathogenicity-related genes–*fliM*, *gumD*, *hrpG*, and *hrpX*–were chosen. The primer sets for all genes were designed for quantitative RT-PCR (qPCR) based on the whole genome sequence of strain PL36 (unpublished information) using Primer3 v4.1.0 (https://primer3.ut.ee/, accessed on 27 June 2025) (Table 1).

### 2.3. Experiment Design and RNA Extractions

**Figure 1:**
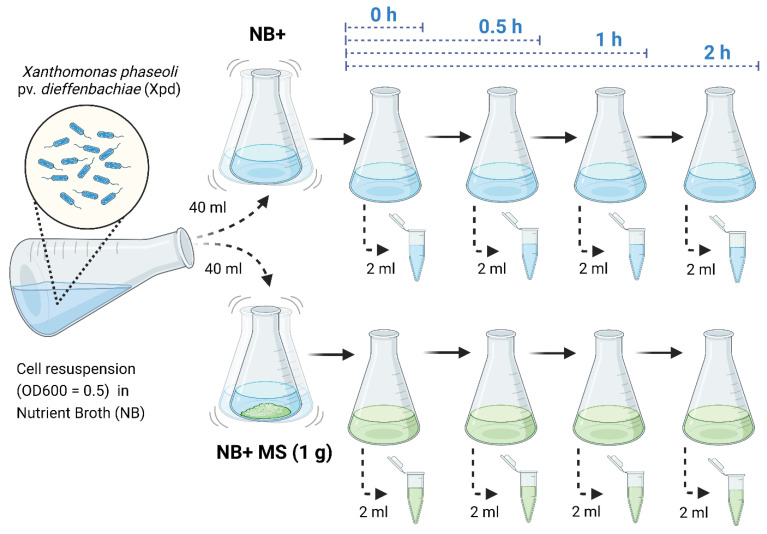
Schematic representation of the time course in vitro assay for the interactions between *Xanthomonas phaseoli* pv. *dieffenbachiae* and *Anthurium*.

The Xpd strain PL36 was cultured on Nutrient agar (NA) media at 28 °C for two days. A single colony was inoculated into 120 mL of Nutrient broth (NB) broth shaking at 200 rpm at 28 °C for 14–18 h to reach OD_600_ = 0.5 as the initial OD. The time course was set at 0, 0.5, 1, and 2 h after incubating PL36 in both NB only (NB+) and NB with 1 g of MS microplant leaf homogenized using liquid nitrogen method (NB + MS) (Figure 1). At each time interval, 2–3 mL of culture was collected and was centrifuged at 14,000× *g* for 1 min at room temperature (RT). The cell pellets were immediately frozen using liquid nitrogen and stored at −80 °C until further RNA extraction. Total RNAs were extracted using rBAC Mini RNA Bacteria Kit (IBI Scientific, Dubuque, IA, USA) following the manufacturer’s instructions, which included DNase I treatment step. The quality and quantity of RNA samples were assessed using NanoDrop 2000 spectrophotometer (Thermo Fisher Scientific, Waltham, MA, USA). RNA degradation and DNA contamination were examined by electrophoresis on a 0.8% agarose gel run at 100 volts for 0.5 h.

### 2.4. Reverse-Transcription and Quantitative RT-PCR (qPCR)

Two step qPCRs were carried out to evaluate the gene expression levels of reference and target genes. One microgram of extracted RNA was reverse-transcribed to cDNA using MMLV reverse transcriptase (Promega, Madison, WI, USA) and random hexamer (Integrated DNA Technologies, Coralville, IA, USA) through heat denatured method. For each qRT-PCR reaction of 20 µL, including 5 µL of 4X UniPLUS Hotstart qPCR, 1 µL of 100 mM DTT, 1 µL of KleeGreen Dye, 1 µL of cDNA template (approximately 10 ng/µL), 1 µL of specific primer pair (listed in Table 1), and appropriate volume of RNase-free water was prepared using UniPLUS RT-qPCR Master Mix (IBI Scientific). The PCR reaction was conducted using Rotor-Gene Q (QIAGEN LLC, Germantown, MD, USA) with the following conditions: UNG incubation at 25 °C for 2 min, RT incubation at 55 °C for 15 min, enzyme activation at 95 °C for 2 min, followed by 40 cycles of amplification at 95 °C for 10 s and 60 °C for 1 min. A melting curve analysis was subsequently performed using Rotor-Gene Q software version 2.3.1 (Qiagen), with a temperature decrease from 99 °C to 80 °C at a ramp rate of 0.2 °C per second, to verify the specificity of amplification products.

### 2.5. Expression Stability of Candidate Reference Genes

The stability of four reference genes was evaluated by pooling four different time point samples, i.e., 0, 0.5, 1, and 2 h after incubating PL36 in both NB only (NB+) and NB with MS plant powder (NB + MS). The PCR amplification efficiency (*E*) and the regression coefficient (R^2^) of the standard curves were estimated using the 10-fold serial dilutions of mixed cDNA samples. Meanwhile, qRT-PCR reactions were carried out for four reference genes in samples from each individual time point, growing in both NB+ and NB + MS media. The relative expression levels of the candidate reference genes were calculated using the threshold cycle (Ct), as determined by the Rotor-Gene Q series software 2.3.1 (Built 49). The expression stabilities of reference genes were analyzed and evaluated using three widely accepted algorithms: standard deviation (SD) and coefficient of variation (CV) in BestKeeper, which assess gene stability based on raw Ct values; the M value in geNorm, which calculates average pairwise variation between genes to identify the most stable candidates; and the average pairwise standard deviation in the ΔCt method, which compares relative expression differences between gene pairs across all samples [20,21,22].

### 2.6. Validation of Reference Genes

Four target pathogenicity-related genes–*hrpG*, *hrpX*, *fliM*, and *gumD*–were used to validate the suitability of the selected reference genes in PL36 cultured with NB + MS. The relative fold changes in gene expressions of each time point sample were calculated using the delta delta Ct (2^–∆∆Ct^) method [24] and normalized using the optimal candidate reference genes. The potential virulence factor with highly gene expression fold change evaluated by comparing samples of NB+ and NB + MS.

## 3. Results and Discussion

### 3.1. Primer Specificity and Amplification Efficiency of Candidate Reference Genes

The quality of extracted RNA samples was evaluated using Nanodrop 2000 spectrophotometer. The ratio of A_260_/A_280_ ranging from 2.15 to 2.19 indicated the high quality of eight RNA samples. The agarose gel confirmed the absence of DNA contamination (Figure S1). The primer specificity of reference genes was validated by the single-peak results obtained from the melting curve analysis, and no primer dimers were formed by RT-qPCR. While setting the threshold as 0.1, the PCR amplification efficiency (*E*) of four reference genes were determined by analyzing standard curves of pooled cDNA time course samples of Xpd strain PL36 cultured in NB+ and NB + MS. The efficiencies ranged from 70.8% to 88.8% in NB+ and from 83.2% to 92.1% in NB + MS (Table S1). However, the values of correlation coefficient (R^2^) of the standard curves were higher than 0.985 for all reference genes.

The *atpD* gene depicted the lowest *E* value in NB+ broth media but the highest *E* value in NB + MS. Although the relatively low *E* values (<90%) of all reference genes presented in the pooled NB+ samples, *atpD* and *gyrB* were regarded as optimal reference genes with *E* > 90% in the pooled NB + MS samples for RT-qPCR amplification. The lower *E* values could result from the higher threshold of Ct value (0.1) used to normalize all reference genes.

### 3.2. Expression Stability

**Figure 2:**
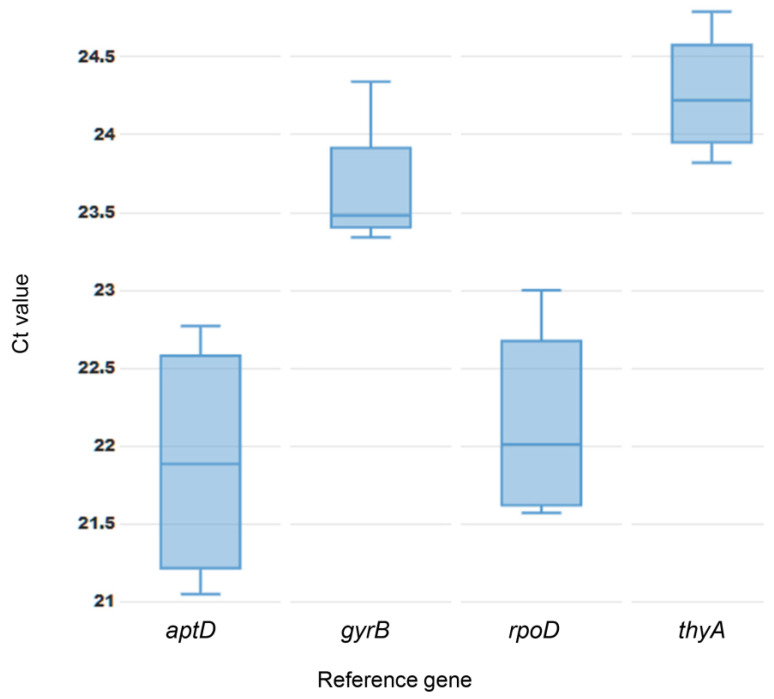
Average Ct value of four time point samples for four candidate reference genes in *Xanthomonas phaseoli* pv. *dieffenbachiae* PL36 strain cultured in NB + MS liquid medium.

Threshold cycle (Ct) value, which is inversely proportional to gene expression abundance, was used to verify the stability of four reference genes by executing statistical applets: BestKeeper, geNorm, and the ΔCt method. The Ct values of four reference genes varied across individual time point samples cultured in NB + MS (Table S2). The mean Ct value from low to high was 21.90 in *atpD*, 22.15 in *rpoD*, 23.66 in *gyrB*, and 24.26 in *thyA* (Table S2, Figure 2).

The BestKeeper, an Excel based tool, was employed to calculate the pairwise correlation analysis on time course samples cultured in NB + MS (Table S2). The more stable candidate displays the lower SD of Ct value [20]. Based on BestKeeper analysis, the order of stability of the four genes was *thyA* (0.31) > *gyrB* (0.34) > *rpoD* (0.53) > *atpD* (0.68) (Table 2 and Table S2).

Meanwhile, the geNorm method assessed the stability of reference genes by calculating M values. Lower M values indicate higher stability; if M ≤ 0.5, it is typically considered stable [21]. The relative quantities of the four reference genes displayed varied expression profiles (Figure S2). The average M value of four reference genes was 0.422 and only M value in *atpD* was higher than 0.5 (Table 2). The ranking of the optimal reference genes based on their stability was: *rpoD* (0.35) > *gyrB* (0.37) > *thyA* (0.45) > *atpD* (0.52) as shown in Table 2. The ΔCt method was performed to obtain the average SD of pairwise ΔCt values in all the reference genes (Table 2; Figure S3). The most stably expressed reference genes were *rpoD* (SD: 0.34) and *gyrB* (SD: 0.37), whereas the least stably expressed genes, *thyA* and *aptD*, had SD of 0.45 and 0.51, respectively (Table 2).

The similar ranking results were obtained using geNorm applet and ΔCt method. The *atpD* was the least stable gene based on the results from all three algorithms, whereas *thyA* was identified as the best choice when assessing using BestKeeper, but not when evaluating with the other two methods. Overall, *rpoD* and *gyrB* ranked in first and second place, respectively, making them the most stable genes and suitable as internal controls for RT-qPCR (Table 2). Considering the amplification efficiency and correlation coefficient of the four reference genes in PL36 growing with NB + MS (Table S1), *gyrB* is the optimal reference gene for gene expression normalization in the Xpd-*Anthurium* interaction.

### 3.3. Expression Profile of Target Genes

**Figure 3:**
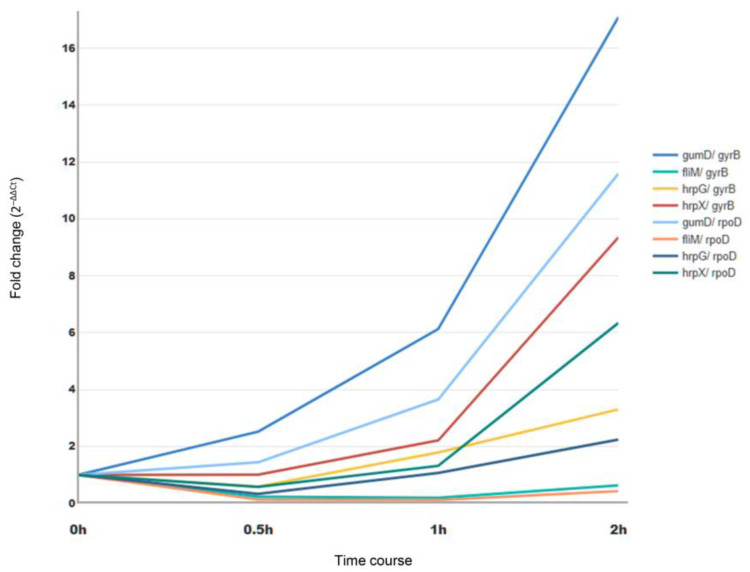
Fold changes in gene expression of four target genes using *gyrB* and *apoD* as internal controls across four time point samples cultured in NB + MS.

The practicality of the optimal reference genes, *rpoD* and *gyrB*, was validated through their use in normalizing the expression of four pathogenicity-related genes–*hrpG*, *hrpX*, *fliM*, and *gumD*– in PL36 cultured with NB + MS medium. The raw Ct data for four target genes at four different time points was normalized using the gene expression of two selected reference genes separately and the expression profiles of each target gene were displayed using the 2^–∆∆Ct^ calculation. The expression profile patterns of each target gene normalized by *rpoD* were similar to those normalized by *gyrB* over the time course; however, the fold changes were shifted (Figure 3).

The encoding genes of both HrpG and HrpX, which control the gene expression of T3SS, T3 effectors, and T2SS substrate encoded genes as regulators [25,26,27], were upregulated after 0.5 h. Interestingly, *hrpX* significantly increased, ranging from 4.23 to 4.81 folds, from 1 h to 2 h samples, whereas *hrpG* exhibited an increase of about 1.85–2.10 folds during the same period (Figure 3). The continually increasing gene expression of *hrpX* and *hrpG* suggest they play important roles on the downstream genes in the HrpG and HrpX-related regulation cascades, which were revealed by the microarray analysis [27].

In addition, the *fliM* gene involved in flagellar biosynthesis was downregulated in 2 h interaction between Xpd-MS (Figure 3). The results might be attributed to signal transmission from HrpG to HrpX and to HrpG’s inhibition of flagellar assembly and chemotaxis within the *hrpG* and *hrpX* regulons [27,28]. Interestingly, RNA-seq analysis in the in vitro system using rice leaf extract showed that the expression of *hrpG* and *hrpX* peaked within 10–15 min, while *fliM* expression was downregulated during the first 10 min and then upregulated afterward [28]. This difference suggests a strategic infection program in which *X. oryzae* pv. *oryzae* rapidly activates T3SS regulators (*hrpG* and *hrpX*) to deliver effector proteins and suppress plant defenses, while initially repressing flagellar genes such as *fliM* to avoid early detection by host immunity, followed by later upregulation to facilitate bacterial colonization and spread.

**Figure 4:**
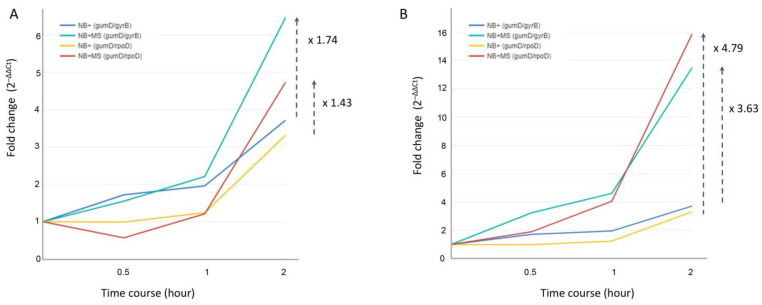
Relative expressions of the *gumD* gene normalized using two reference genes, *gyrB* and *rpoD*, in NB+ and NB + MS liquid broths. (**A**) The gene expression was calculated using the 0 h-NB+ sample as the baseline to determine fold changes for both sets. (**B**) The gene expression of the 0 h-NB+ and 0 h-NB+MS samples were calculated as the individual baselines in NB+ and NB + MS sample sets. Dashed arrows indicate the fold changes in gene expression between NB+ and NB + MS samples at the 2 h time point.

The expression profile of the *gumD* gene, which encoded the pentasaccharide repeating unit of xanthan [29], showed the highest upregulation in NB + MS samples during the 2 h incubation compared to that of other target genes (Figure 3). In the previous study, the *X*. *campestris* pv. *campestris gumD* knockout mutant displayed a reduced virulence during infection on broccoli leaves [30]. Similarly, the *X*. *oryzae* pv. *oryzae gumD* knockout mutant showed a significant reduction in lesion size, decreasing to about 11% on the susceptible rice cultivar IR24 [31]. Therefore, the elevated gene expression suggests that the *gumD* gene is likely one of the key genes influencing pathogenicity in the Xpd-*Anthurium* pathosystem. To determine whether the highest gene expression of *gumD* was induced by the addition of *A. andreanum* MS microplant powder, a comparison of *gumD* gene expression profiles, normalized using the two optimal reference genes—*gyrB* and *rpoD*—was performed on time course samples cultured with NB+ and NB + MS, respectively (Figure 4). Using the 0 h-NB+ sample as a baseline, the results demonstrated differential gene expression after 1 h, and the *gumD* gene, normalized by *gyrB* and *rpoD*, respectively, was upregulated about 1.74- and 1.43-fold in the 2 h-NB+MS sample compared to the 2 h-NB+ sample (Figure 4A). Notably, the *gumD* gene expression profiles differed depending on whether the 0 h-NB+ and 0 h-NB+MS samples were independently used as the baseline for each time course set (Figure 4B). The differences in fold changes were more than twofold when calculated using different baselines, indicating that the gene expression changes occurred immediately after the addition of MS microplant powder. The *gyrB* gene demonstrated greater stability than the *rpoD* gene due to its consistent gene expression profile (Figure 4). Despite the varied gene expression levels, the *gumD* gene, involved in the biosynthesis of xanthan gum, differentially regulated pathogenicity in the interactions between Xpd and the susceptible *A. andreanum* cultivar MS.

## 4. Conclusions

This study aimed to select the optimal reference gene for *X. phaseoli* pv. *dieffenbachiae* while interacting with the host plant *A. andreanum*. The in vitro system using anthurium leaf extract successfully activated the tested pathogenicity-related genes. The BestKeeper, geNorm, and delta Ct methods summarized *rpoD* and *gyrB* as the most stable housekeeping genes during the initial time course of the in vitro pathosystem. These two optimal reference gene candidates were able to assess the similar gene expression patterns of four pathogenicity-related target genes. The flagellar-related gene, *fliM*, was downregulated within 2 h. On the other hand, the activation of *hrpX* and *hrpG* was detected after 0.5 and 1 h, respectively, with their gene expressions levels continually upregulated. The highest gene expression of *gumD* was observed at the 2 h time point in the NB + MS samples, while no significant increase was observed in the non-treatment samples, suggesting its importance as a virulence determinant. To our knowledge, this is the first study to identify reference genes for studying the interactions between Xpd and *A. andreanum*. The identified ideal gene normalizers are suitable for the further validation of transcriptome analysis using RNA-seq technology.

## Figures and Tables

**Table 1:** The primer sets of reference and target genes designed in the study.

Gene Candidate	Gene Name	Primer Sequence (5′-3′)	Amplicon Size (bp)	Tm (°C)
Reference genes (RG)	*atpD*	atpD_QF: GCTGAGCGAAGAAGACAAGC atpD_QR: GTCCTTCAGCGAGACGTACT	124	62
	*gyrB*	gyrB_QF: TGACCGACGAACAAAACACC gyrB_QR: CATCGCCGATATACATGCCG	117	60
	*rpoD*	rpoD_QF: ATGAAAATCGCCAAGGAGCC rpoD_QR: TGATGTTGGTGGTGTTGTCG	124	60
	*thyA*	thyA_QF: AAGCCGTACCTGGAGTTGTT thyA_QR: GGAAAGCCGTCGTTGAGATC	125	60
Target genes (TG)	*fliM*	filM_QF: TGGACGTGGACTTCGAGTAC filM_QR: GAATACGGCAGGGTGATGTG	145	60
	*gumD*	gumD_QF: TCCTGAACCATCTGCGTACC gumD_QR: GTTACGGCTCAGGTAGTGGT	101	60
	*hrpG*	hrpG_QF: ATCGGTGTTTCTGTTGACGC hrpG_QR: GAAGCTCCAGTTCCTCGGAA	107	60
	*hrpX*	hrpX_QF: ACTGCAACATCTCCAACAGC hrpX_QR: ATACGCATCTTCGGCCTCTT	133	60

**Table 2:** Final ranking and statistical comparison of four reference genes in *X. phaseoli* pv. *dieffenbachiae* PL36 cultured in NB + MS medium across four time points by BestKeeper, geNorm and delta Ct methods.

Reference Gene	BestKeeper			geNorm			ΔCt		Final Rank
	SD [±Ct]	CV [% Ct]	Rank	M Value	CV	Rank	SD*	Rank	
*atpD*	0.68	3.12	4	0.52	0.28	4	0.51	4	4
*gyrB*	0.34	1.44	2	0.37	0.15	2	0.37	2	2
*rpoD*	0.53	2.38	3	0.35	0.10	1	0.34	1	1
*thyA*	0.31	1.29	1	0.45	0.21	3	0.45	3	3

SD [±Ct]: the standard deviation of the Ct; CV [% Ct]: coefficient of variance expressed as a percentage of the Ct value; M value: the measure of a gene’s expression stability; CV: coefficient of variance in geNorm; SD* (ΔCt*): mean of pairwise ΔCt values in ΔCt method.

## Data Availability

The original contributions presented in this study are included in the article. Further inquiries can be directed to the corresponding author.

## Supplementary Materials

The following supporting information can be downloaded at: https://www.mdpi.com/article/10.3390/mps8040072/s1, Figure S1. Agarose gel result of RNA samples cultured in NB+ and NB + MS broth media across different time points. L denoted a 100 bp ladder. Figure S2. Expression stability of four candidate reference genes (*atpD*, *gyrB*, *rpoD*, and *thyA*) analyzed using geNorm. The relative quantities (±SEM) of the reference genes were evaluated across four time point samples of NB + MS inoculated with *Xanthomonas phaseoli* pv. *dieffenbachiae* PL36. SEM: standard error of the mean. Figure S3. Box plot shows the pairwise comparisons of delta Ct (ΔCt) among four candidate reference genes in *Xanthomonas phaseoli* pv. *dieffenbachiae* cultured in NB + MS broth medium. The average ΔCt value across samples of four time points were analyzed for *atpD* (A), *gyrB* (G), *rpoD* (R), and *thyA* (T) genes. The lines within boxes indicate the median values, the boxes indicate the interquartile range (25th to 75th percentile), and the whiskers indicate the full range of the data. Table S1. Parameters of the standard curve derived from qRT-PCR using 10-fold serial dilutions (10–0.01 ng/µL) of pooled cDNA from *Xanthomonas phaseoli* pv. *dieffenbachiae* (Xpd) strain PL36 cultured in NB+ and NB + MS broth media. Table S2. Expression stability of candidate reference genes in PL36 cultured in NB with MS plant powder analyzed using BestKeeper.
