# Supplementary material for: Optimal Reference Gene Selection and Potential Target Gene Identification During Xanthomonas phaseoli pv. dieffenbachiae–Anthurium andreanum Infection
Source: Methods Protoc. 2025 Jul 4;8(4):72. doi: 10.3390/mps8040072 (PMC12286264; doi:10.3390/mps8040072)
Supplement: Supplementary file 1 [file mps-08-00072-s001.zip › Table S1-S2.pdf]

Table S1. The parameter of standard curve derived from qRT-PCR using 10-fold serial dilutions (10-0.01 ng/µl) of pooled cDNA of *Xanthomonas phaseoli* pv. *dieffenbachiae* (Xpd) strain PL36 culturing in NB+ and NB+MS broth media.

**Gene**	**Xpd in NB+**		**Xpd in NB+MS**	
	**PCR efficiency (*E*)**	**Correlation coefficient (R^2)**	**PCR efficiency (*E*)**	**Correlation coefficient (R^2)**
atpD	70.8%	0.994	92.1%	0.996
gyrB	84.1%	0.998	90.4%	0.997
rpoD	88.8%	0.996	85.8%	0.990
thyA	86.4%	0.993	83.2%	0.985

NB+: NB broth medium only.

NB+MS: NB broth medium adding 1 gram of MS ground plant powder.

Optimized $R^2 > 0.98$

Table S2. The expression stability of candidate reference genes of PL36 culturing in NB with MS plant powder analyzed by BestKeeper.

**Reference Gene**	**Time course (hour)**				**Statistical parameters**					
	**0 [Ct]**	**0.5 [Ct]**	**1 [Ct]**	**2 [Ct]**	**GM [Ct]**	**AM [Ct]**	**Min [Ct]**	**Max [Ct]**	**SD [±Ct]**	**CV [%Ct]**
atpD	22.39	21.05	21.38	22.77	21.89	21.90	21.05	22.77	0.68	3.12
gyrB	23.47	23.49	23.34	24.34	23.66	23.66	23.34	24.34	0.34	1.44
rpoD	22.35	21.67	21.57	23	22.14	22.15	21.57	23	0.53	2.38
thyA	24.08	24.36	23.82	24.79	24.26	24.26	23.82	24.79	0.31	1.29

Ct: Crossing point, in terms of Ct value
GM: Geometric Mean
AM: Arithmetic Mean
Min: Minimum
Max: Maximum
SD: Standard deviation
CV: Coefficient of variance
