# Supplementary figures and images for: Optimal Reference Gene Selection and Potential Target Gene Identification During Xanthomonas phaseoli pv. dieffenbachiae–Anthurium andreanum Infection
Source: Methods Protoc. 2025 Jul 4;8(4):72. doi: 10.3390/mps8040072 (PMC12286264; doi:10.3390/mps8040072)

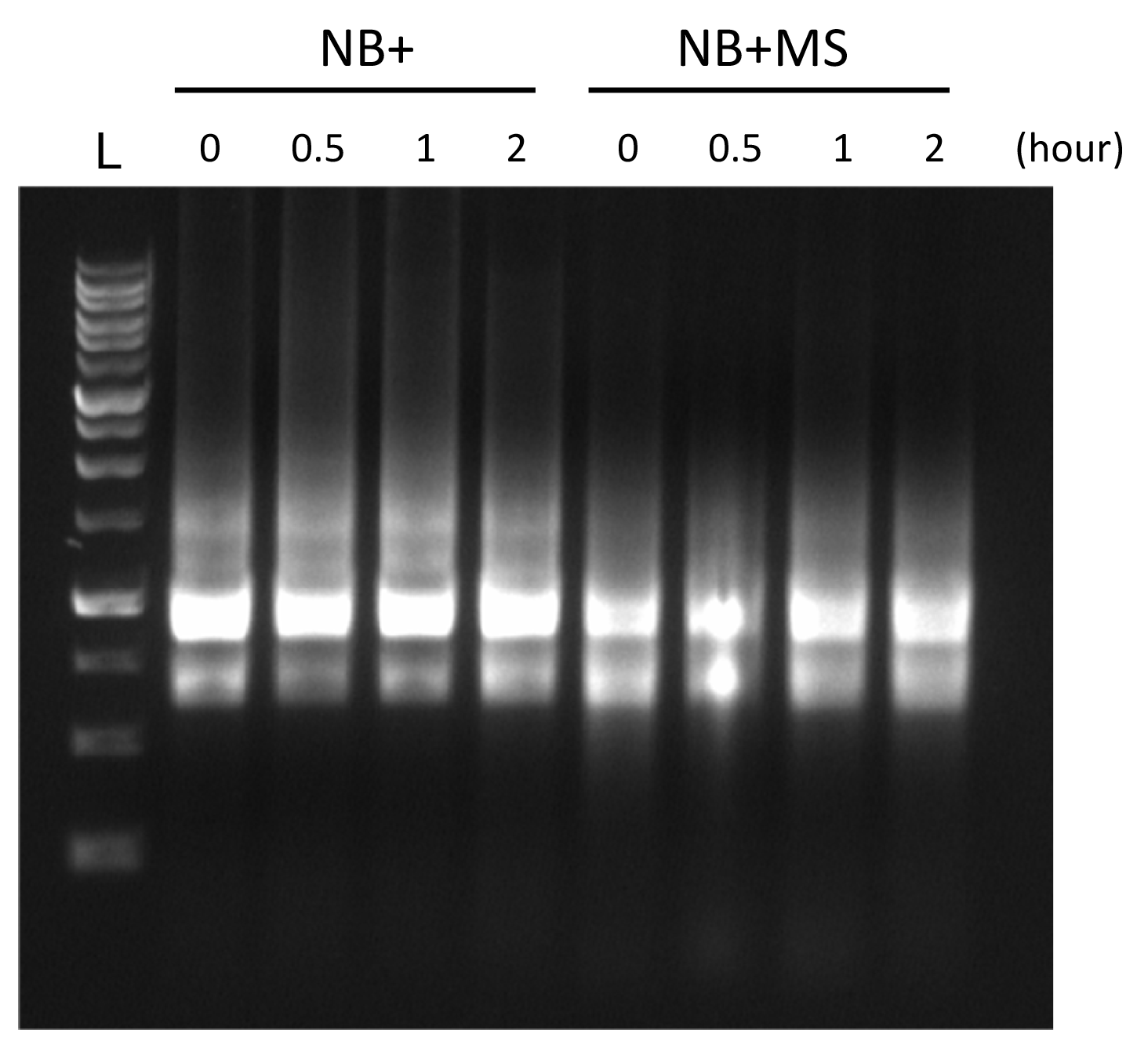

Supplement: Supplementary file 1 [file mps-08-00072-s001.zip › Figure S1.tif]

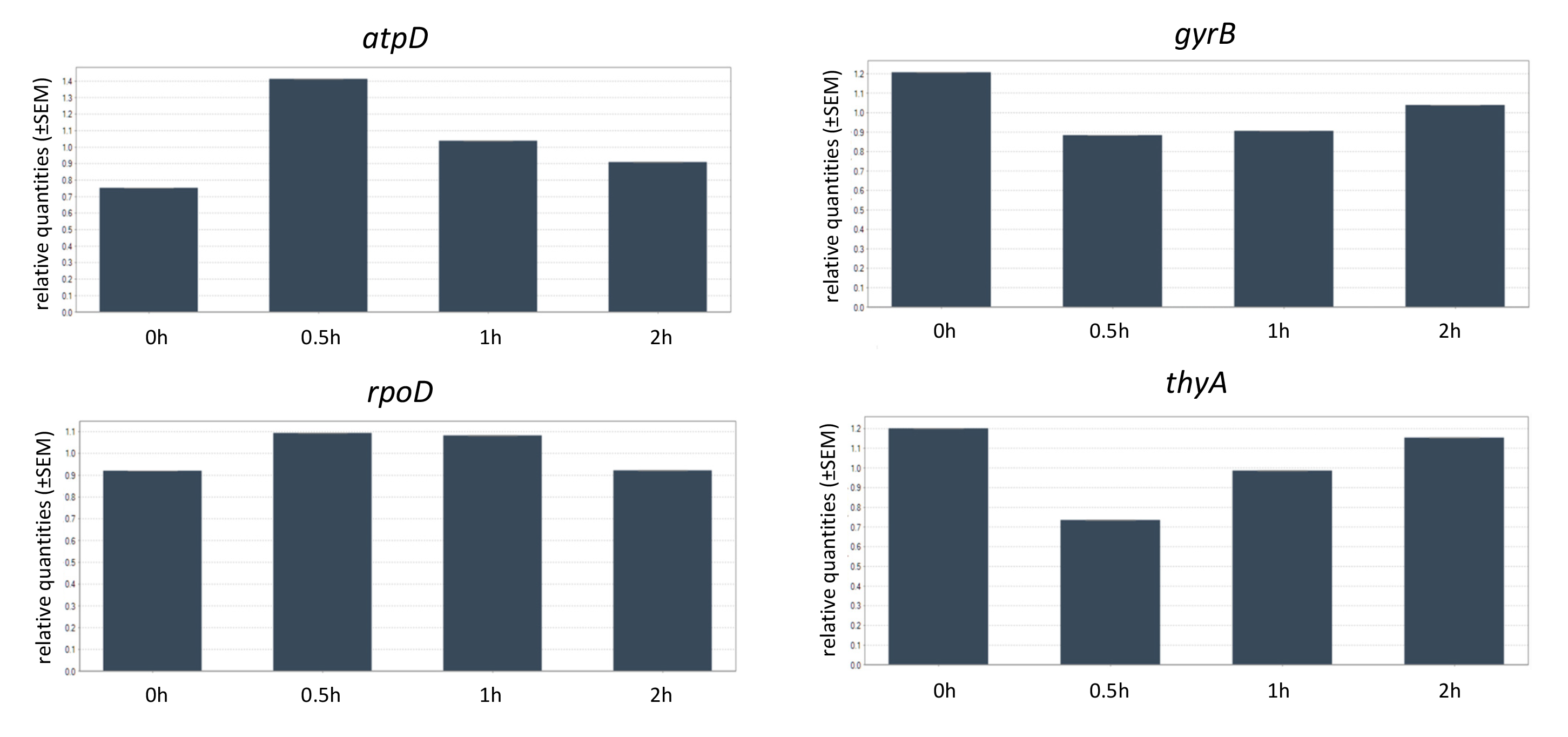

Supplement: Supplementary file 1 [file mps-08-00072-s001.zip › Figure S2.tif]

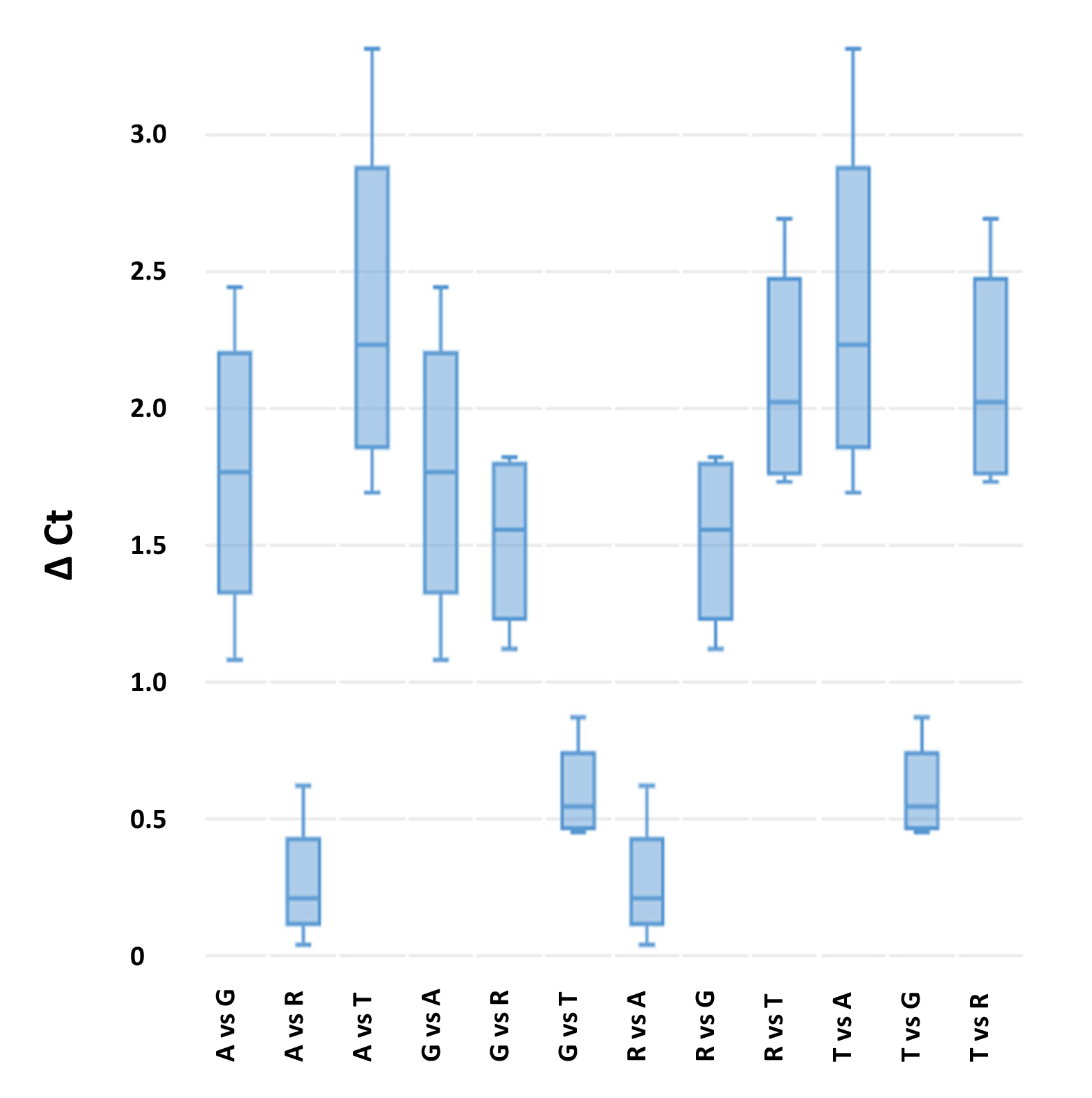

Supplement: Supplementary file 1 [file mps-08-00072-s001.zip › Figure S3.tif]
